# Assessing the Effects of Nature on Physiological States Using Wearable Technologies

**DOI:** 10.3390/ijerph19031231

**Published:** 2022-01-22

**Authors:** Dannie Fu, Natalia Incio Serra, Hubert Mansion, Emilia Tamko Mansion, Stefanie Blain-Moraes

**Affiliations:** 1 Department of Biomedical Engineering, McGill University, Montreal, QC H3A 2B4, Canada; dannie.fu@mail.mcgill.ca; 2 School of Physical & Occupational Therapy, McGill University, Montreal, QC H3G 2M1, Canada; natalia.incioserra@mail.mcgill.ca; 3 L’Université Dans la Nature, Montreal, QC H1V 1H6, Canada; hubert.tlmvdn@gmail.com (H.M.); emilia@unature.org (E.T.M.)

**Keywords:** forest bathing, autonomic nervous system, profile of mood states, wearable technology, forest therapy

## Abstract

Nature therapy and forest bathing (FB) have been shown to have quantifiable positive effects on human health, but the physiological effects of a guided interactive nature activity remain unexplored. Autonomic nervous system responses to a guided nature walk (Nature Break) were assessed through the continuous measurement of the electrodermal activity (EDA), fingertip temperature, and heart rate (HR) of *n* = 48 participants, using a wearable sensor. Psychological distress was assessed before and after the activity using the Profile of Mood States (POMS) for *n* = 38 (24 females, 14 males, mean age = 43.55 ± 11.61 years) participants. The negative dimensions of POMS decreased and the positive (vigor) dimensions increased following a Nature Break. Significant differences were found across all of the physiological features, with some differences occurring between the morning and afternoon groups and between different days. The participants’ mean HR decreased throughout the Nature Break. Our results suggest that interactive nature activities have positive psychological benefits and demonstrate the feasibility of using wearable sensors to monitor physiological responses in a naturalistic forest bathing activity.

## 1. Introduction

In 2018, Northern America was the most urbanized geographic region in the world with 82% of its population living in urban areas. Globally, 55% of the world’s population was living in urban areas in 2018 with this number projected to reach 68% by 2050 [1]. Rapid urbanization poses many socio-economic, environmental, and psychological challenges, such as increased levels of crime, air and water pollution, and the psychological stressors that are associated with higher levels of density and diversity of cities. The environmental particulate matter that is associated with decreased air quality may be related to cardiovascular deaths and asthma and noise exposure may be associated with hearing impairment, hypertension, and ischemic heart disease [2].

Over the past few decades, there has been growing interest surrounding the impacts of natural environments on human health and well-being. In 1982, the term shinrin-yoku, also known as forest bathing (FB), was coined by the Ministry of Agriculture, Forestry and Fisheries of Japan to describe the practice of immersing oneself in a forest environment using all five senses in order to improve one’s mental and physical health [3,4,5]. The accumulating evidence has demonstrated that exposure to nature has quantifiable positive effects on the psychological and physiological health of human beings [3,5,6,7]. In particular, the act of immersing oneself in a forest has been associated with decreased blood pressure in comparison to immersion in urban environments [4,8]; significant decreases in physiological biomarkers of stress such as salivary cortisol and alpha-amylase [9,10]; and improvements in psychological measures of tension, anger, fatigue, anxiety, confusion, and depression [4,11,12,13].

Research in this field has been translated directly into various health initiatives, as FB and nature walks have become increasingly popular as methods of promoting active lifestyles and improving overall wellness [14] and have started being prescribed by doctors and recommended by federal, provincial and local associations. On the global scale, Health Parks Healthy People (HPHP) is a movement that is working alongside many large groups, such as the National Park Service in the United States and Ontario Parks in Canada, to advocate the physical and mental benefits of nature. For example, Mood Walks is a program led by the Canadian Mental Health Association, Ontario, that promotes nature walks to improve physical and mental health.

Research to date has provided psychological and physiological evidence regarding the positive impacts of FB and nature walks. To date, most physiological studies have primarily assessed the effects of natural environments on cardiovascular measures, such as heart rate, heart rate variability (HRV), and blood pressure [3,4,5,12,15,16,17,18]; neuroendocrine or metabolic indexes, such as cortisol and adrenaline [5,19,20,21]; or immune and inflammatory indexes, such as natural killer (NK) cells and tumor necrosis factor-alpha (TNF-α) [5,22,23,24]. Few studies have assessed the physiological effects of natural environments on electrodermal activity (EDA) or peripheral skin temperature, which directly reflect sympathetic and parasympathetic nervous system activity and have been reliably connected to an individual’s mental and emotional state [25,26,27,28].

Further, only a few studies have investigated purposeful engagement with the environment during a nature therapy session or walking session [29,30,31]. Duvall (2013) looked at the impact of cognitive engagement strategies, such as focusing on a sense (e.g., noticing different sounds) or taking on a new role (e.g., imagining they are looking for inspiration as an artist), on well-being and perception of the environment during outdoor walking sessions. They found that only those in the engagement condition became more satisfied with multiple aspects in their walking environment, such as general appearance, nature sounds, and the variety of things to look at [30]. Furthermore, the engagement condition was associated with self-reported significant increases in attention and decreased feelings of frustration [29,30]. Similarly, Korpela et al. (2017) found that focused attention on interactions with nature was associated with mood enhancement and self-reported positive restoration [31].

Building upon these studies, we aimed to assess the in-situ effects of a 120-min guided Nature Break activity that encouraged participants to deliberately engage with their environment. In order to characterize the effects of this activity on an individual’s physiological state, we recorded three signals that reflect sympathetic and parasympathetic activity: EDA, fingertip temperature, and HR. We monitored these signals using a wearable sensor and mobile application in order to obtain moment-by-moment measures of physiological reactions that could be correlated with the specific activities in the forest. We also assessed the effect of the Nature Break activity on participants’ self-reported mood states using the POMS questionnaire. We hypothesized that a Nature Break would psychologically reduce negative affect and increase positive affect, in accordance with previous studies. We also hypothesized that the different activities in the forest would induce distinct and dissociable physiological response profiles in Nature Break participants.

## 2. Materials and Methods

### 2.1. Description of the Nature Break Activity

#### 2.1.1. L’Université dans la Nature

L’Université dans la Nature (UdN) is a non-profit organization that is located in Montreal, Canada. UdN’s vision is to create stronger and more sustainable communities and economies through solutions that are inspired by nature. The mission of L’UdN is to equip the decision-makers of today and tomorrow (e.g., university students) with reliable and sustainable methods of decision-making, stimulating creativity and innovation, and preventing stress through encounters with nature.

#### 2.1.2. Nature Break

Nature Break is a guided immersion event in the forest that lasts approximately 120 min. The guide educates the participants about the neuroscientific findings of the impacts of nature on human health and also aims to catalyze a personal connection with nature. This activity seeks to generate awareness of the changes in mental and physiological state that are induced by anchoring the reasons for these changes in a concrete experience with measurable effects.

Nature Break was modelled on scientific research conducted in Scandinavia, the United States and Southeast Asia [32,33,34]. This non-sporting and non-doctrinal activity is framed by sensory exercises and a dissemination of scientific knowledge about the benefits of nature that are within everyone’s reach.

Nature Break is comprised of five principal segments in the forest, which incorporate various themes surrounding the five senses (e.g., touch, sight, hearing, etc.). Individuals are invited to perform exercises that stimulate their senses in each segment; all segments were designed to guide and stimulate the participant’s perception and sensory experience of the forest. See Table 1 for a detailed description of the segments and exercises.

### 2.2. Participants

A total of 58 individuals participated voluntarily in this study through UdN’s Nature Break program. Thirty-eight participants from two days of data collection completed the POMS questionnaire (*n* = 38, 24 females, 14 males, mean age = 43.55 ± 11.61 years); and 48 participants across all of the days of the data collection period provided their physiological data for analysis. All of the participants were fluent in English and French.

### 2.3. Study Site

The study was conducted in the autumn season on a private woodlot in Barnston Ouest, Quebec, which is located in the Great Lakes–St. Lawrence forest region in Canada. This forest region is characterized by a mix of deciduous and coniferous trees, including red pine (*Pinus resinosa*), eastern white pine (*Pinus strobus*), and yellow birch (*Betula alleghaniensis*), as well as some boreal species, such as the American beech (*Fagus grandifolia*) (Information from https://www.sfmcanada.org/en/canada-s-forests1 accessed on 26 January 2021. The private woodlot was located away from traffic and the city. The Nature Break activity took place in the forest where participants were immersed in the forest setting and could not hear or see traffic or buildings. Meteorological data was collected from a nearby station in Coaticook (latitude: 45°09′00.000″ N, longitude: 71°48′00.000″ W).

### 2.4. Procedure

The study was conducted on three days during the last two weeks of September, 2020. The maximum and minimum temperatures were 11 °C and −3.1 °C, 26.5 °C and 12.5 °C, and 23.8 °C and 17.1 °C for days 1 through 3, respectively. Each day accommodated a group of ten participants in the morning, followed by another group of ten participants in the afternoon. Upon arrival at the site, participants were greeted by the co-founder of the Nature Break program who gave them a brief overview of the study and instructed them to fill out both the informed consent form and the POMS for the pre-forest assessment of their psychological state. Subsequently, we introduced the physiological monitoring technology to the participants, showing them how to wear the sensor, what data was being recorded, and how to use the smartphone application. Participants then donned the physiological sensor on their dominant hand and kept the paired smartphone in their purse, pocket, or held in their hand for the remainder of Nature Break. Baseline physiological measures were obtained from when the participant put the sensor on to just prior to starting the activity.

We briefly reiterated the goals and content of the Nature Break and the study to the group of participants, prior to proceeding to the forest. The Nature Break activity consisted of eight segments (Table 1). After the activities in the forest, the participants were led to a field where they filled out the POMS for the post-forest assessment of their psychological state. The Nature Break’s guides led a discussion with the entire group about their experiences in the forest.

### 2.5. Data Collection

#### 2.5.1. Psychological Measurements

The Profile of Mood States (POMS) was used as a measure of psychological distress. The original tool consists of 30 adjectives which collectively measure six effective dimensions: tension-anxiety (T), depression-dejection (D), anger-hostility (A), fatigue-inertia (F), vigor-activity (V), and confusion-bewilderment (C). Each adjective is rated using a 5-point scale from 0 to 4. Our study uses an adapted version of the POMS, consisting of 18 adjectives [36].

#### 2.5.2. Physiological Measurements

The autonomic nervous system (ANS) is composed of the parasympathetic and sympathetic branches, otherwise known as the “rest or digest” and “fight or flight” systems. Typically, excitation of the sympathetic nervous system and inhibition of the parasympathetic nervous system occurs in response to states such as anxiety and stress [37], while the opposite may occur during states of relaxation [38,39]. Changes in autonomic nervous system (ANS) signals, such as EDA, HR, and skin temperature, are connected to internal emotional or mental states [40,41].

Three physiological signals reflecting ANS activity were collected from a wearable device called the Triple-Physiology Sensor (TPS) (Thought Technology Ltd.©, Montreal, Quebec, Canada). The TPS is worn on the fingertip and secured with Velcro wrap. It collects three physiological signals: (1) electrodermal activity (EDA), (2) skin temperature, and (3) blood volume pulse (BVP), which is used to derive heart rate (HR) and heart rate variability (HRV).

##### Electrodermal Activity (EDA)

Electrodermal activity (EDA) reflects sympathetic innervation of the ANS and has long been used as an indicator of emotional arousal to external or internal stimuli, such as emotional thoughts, memory recall, or novel, startling, or threatening stimuli [38,42]. These stimuli may evoke electrodermal reactions (EDRs) which appear as transient peaks in the EDA signal.

##### Fingertip Skin Temperature

Fingertip temperature is often used as a measure of sympathetic activity but it is dependent on the subject’s body temperature. When the initial fingertip temperature is above 33.2 °C, sympathetic innervation induces vasoconstriction causing less heat to be radiated from the surface; however, below this temperature, vasoconstriction is not possible and vasodilation occurs in response to the same sympathetic stimulus [38,42]. Fingertip temperature has been shown to decrease in response to stimuli such as sudden noises, fear, pain, and mental stimulation (such as motor imagery), and to increase during mental relaxation [38,42,43].

##### Heart Rate (HR)

The heart is innervated by both the sympathetic and parasympathetic branches of the ANS. Heart rate has been shown to decrease when an individual’s attention is focused on the external environment and when sensory information gathering is high, it has been shown to increase during internal focus or when the sensory input is dampened [38]. Higher HR is also associated with elevated states of stress and anxiety [44,45].

### 2.6. Data Analysis

The difference in psychological and physiological measures was assessed before and after the Nature Break activity. Changes in the physiological signals were also assessed across all of the segments of the activity that took place within the forest.

#### 2.6.1. Psychological Measures Analysis

For the pre-post Nature Break psychological assessment, the mean POMS score for each dimension was taken across all participants (*n* = 38). The pre-post significance was computed using Wilcoxon signed-rank paired *t*-tests. The results were tested against the Bonferroni-Holm adjusted alpha levels of 0.05 and 0.01.

#### 2.6.2. Physiological Measures Analysis

First, each of the ANS signals were pre-processed in order to remove non-physiological artifacts by applying smoothing filters (1D median filter, moving average filter), cubic spline interpolation, and modality specific filters. A one Euro filter was applied to the EDA signal; an exponential decay filter was applied to the skin temperature signal; and a cubic smoothing spline was applied to the HR signals [46].

**Figure 1:**
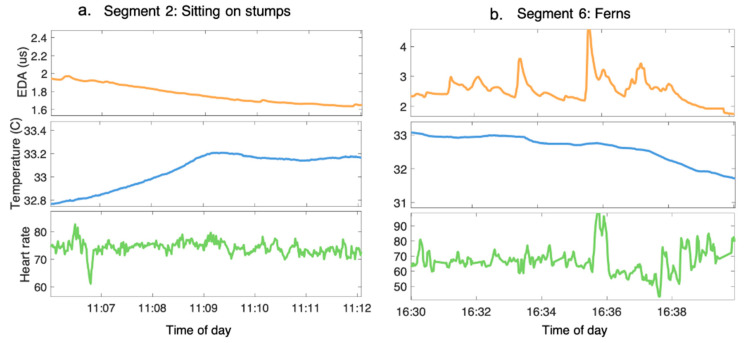
Examples of pre-processed EDA, skin temperature, and HR signals during activity segments in the forest. (**a**). The participant’s signals during segment 2 when they were sitting in a circle on tree stumps, listening to the guide talk. (**b**). The participant’s signals during segment 6, when they were sitting in a bed of ferns. During segment 6, participants were asked to focus on various sensory stimuli, such as sound and the patterns on the leaves.

**Figure 2:**
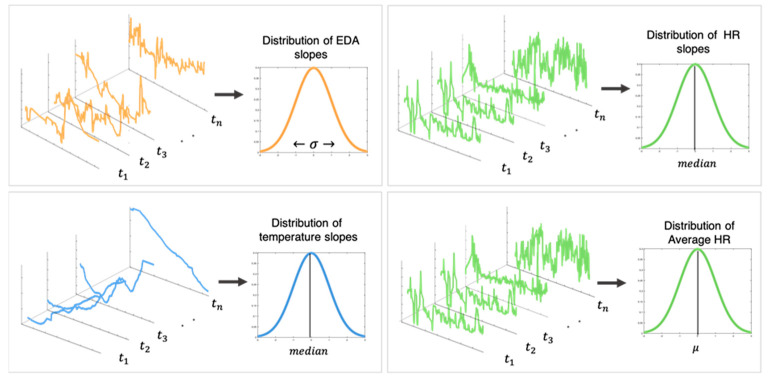
The average slopes of the EDA, HR, and skin temperature were computed across 60 s sliding windows in each forest activity segment. Features extracted for each forest activity segment: standard deviation of the EDA slopes, median of the temperature and HR slopes, mean HR.

The signals were then segmented by forest activity and the following features were extracted [47]: (1) The standard deviation of the slopes of the EDA was used to determine the presence or absence of EDRs; (2) The median of the slopes of the skin temperature was used to determine its general increasing/decreasing trend; and (3) The mean of the slopes of the HR was used to determine its general increasing/decreasing trend. Figure 1 is an example of the physiological signals that were derived from two of the forest activity segments; Figure 2 illustrates the features that were extracted for each physiological signal from each forest activity segment.

The physiological measures were first analyzed across all of the participants, not taking into account the effects of the time-of-day or ambient temperature. We then conducted two control analyses in order to determine the effects of the ambient temperature and time-of-day. We divided the analysis into day 1 participants (*n* = 16) and day 2 and 3 participants (*n* = 33) due to the significant difference in the ambient temperatures between these two groups [38,42]. Kistler et al. (1998) reported that the sympathetic stimulation of fingertip temperature is dependent on the overall body temperature. Across day 1 participants, the average fingertip temperature was 18.8 °C, whereas for day 2 and 3 participants, the average was 30.0 °C. In this paper, we will refer to day 1 and the day 1 participants as a “cool day” and the “cool day participants”, and days 2 and 3 and their respective participants as “warm days” and the “warm day participants”.

We assessed the effect of the time-of-day based on whether the participants were in the morning or afternoon group. Day 2 and 3 participants had their associated POMS scores taken account in the statistical model (day 1 participants did not have psychological data collected to be included in this analysis).

#### 2.6.3. Determining the Physiological Effect of Nature Break

To determine if there was a significant difference in the physiological signals that were recorded before and after the Nature Break, a paired samples *t*-test was used for the normally distributed physiological features and a Wilcoxon signed-rank test was used for the non-normally distributed features. A mixed model was used to assess the significance differences across the forest activity segments, with the segment as a fixed effect. A mixed-model approach was selected in order to take into account the missing data that occurred due to technical difficulties or unclean data.

##### Control Analysis 1: Ambient Temperature

The participants were divided into cool day (day 1) and warm day (day 2, 3) groups in order to take into account the significant difference in ambient temperatures between these days. The pre-post analysis and the analysis across the forest activity segments followed the procedure that was described in Section 2.6.3; morning and afternoon group and pre-forest POMS scores were not considered in this portion of the analysis.

##### Control Analysis 2: Time of Day

For both groups, a paired samples *t*-test or Wilcoxon signed rank test was used to determine if there was a significant difference before and after the Nature Break for each physiological signal. Independent t-tests were used on the pre-post difference in order to assess the difference between the morning and afternoon groups. Welch’s *t*-test was used when the variances were unequal and a Mann-Whitney U test was used for the non-normally distributed features. We performed an Analysis of Covariance (ANCOVA) in order to assess whether the means between the morning and afternoon groups were significantly different post-forest, adjusting for the pre-forest measurements. A mixed model was used in order to assess the significance differences across the forest activity segments, with segment and time-of-day as fixed effects. For the day 2 and 3 participants analysis, the ANCOVA also adjusted for pre-forest positive and negative POMS scores and the mixed model accounted for the pre-forest rounded positive and negative POMS scores.

The *p*-values from the multiple comparisons between morning and afternoon groups at each forest activity segment, as well as multiple comparisons between the segments, were adjusted using the Bonferroni method.

## 3. Results

### 3.1. Forest Bathing Increases Positive Mood States and Decreases Negative Mood States

**Figure 3:**
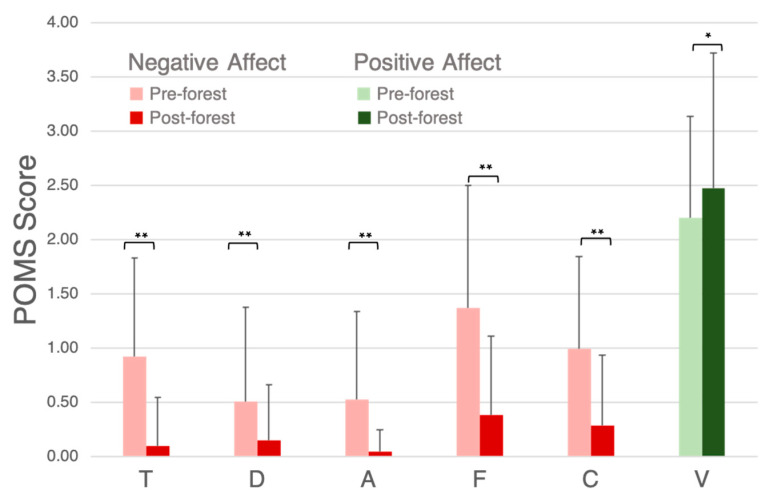
POMS results pre-forest and post-forest. T: tension and anxiety, D: depression and dejection, A: anger and hostility, F: fatigue, C: confusion, V: vigor. Significant differences are marked with * *p* < 0.05 and ** *p* < 0.01.

The average POMS scores for the negative mood states significantly decreased following the Nature Break (*p* < 0.01), while the average POMS score for the positive mood state (V) significantly increased following the Nature Break (*p* < 0.05) (Figure 3).

### 3.2. Purposeful Engagement with the Forest Induces Different Physiological Response Profiles

**Figure 4:**
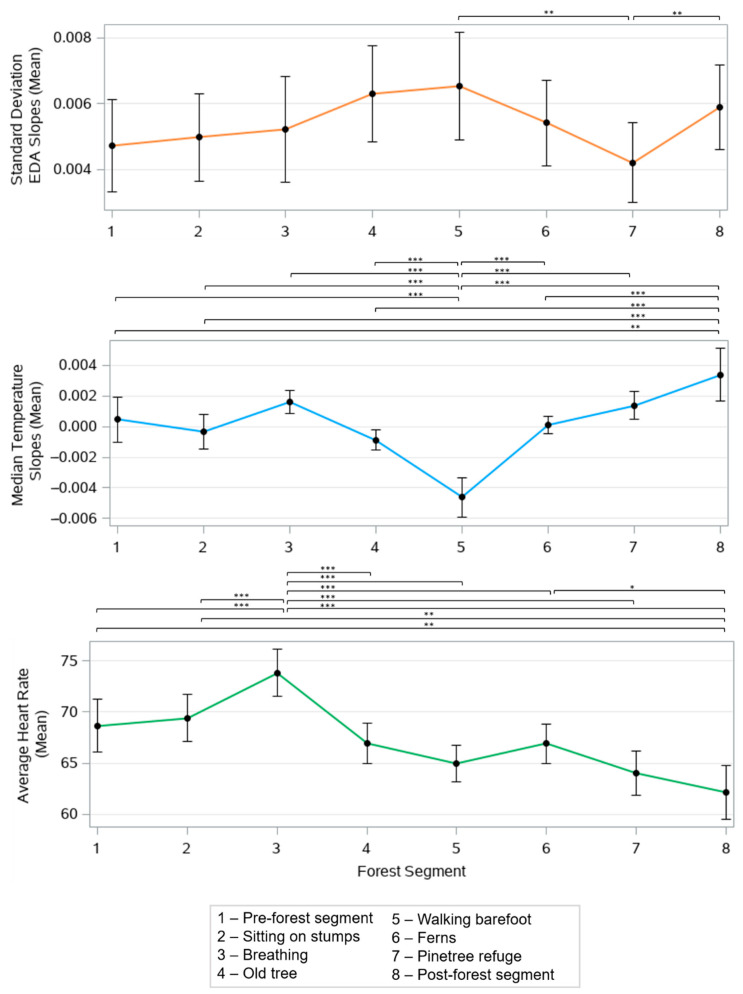
Effect of forest bathing on the mean standard deviation of the EDA slopes, mean of the median temperature slopes, and mean average heart rate across all forest activity segments. * *p* < 0.05, ** *p* < 0.01, *** *p* < 0.001, Bonferroni adjusted *p*-values.

The FB segments had significant effects on all of the recorded physiological features, including the mean standard deviation of the EDA slopes (*p* < 0.001 un-adjusted), mean of the median skin temperature slopes (*p* < 0.001 un-adjusted), and mean of the average HR values (*p* < 0.001 un-adjusted). Figure 4 shows the mean value with a 95% confidence interval of these three measures across all of the segments and the Bonferroni adjusted *p*-values.

The participants experienced the largest sympathetic arousal, as manifested by their EDA, during the old tree (segment 4), barefoot walking (segment 5), and post-forest (segment 8) activity segments, and the lowest sympathetic arousal during the pine tree refuge segment (segment 7). The pine tree refuge segment (segment 7) demonstrated the largest difference to the barefoot walking and the post-forest activity segments (*p* < 0.01), likely due to the various stimulating activities during the latter two segments.

Skin temperature had the largest rate of increase during the breathing activity (segment 3), pine tree refuge (segment 7), and post-forest (segment 8) activity segments, likely indicating relaxation. This feature had the largest rate of decrease during the barefoot walking activity (segment 3), likely reflecting focus and attention. The barefoot walking activity demonstrated a significantly lower skin temperature slope than all of the other segments, with a significance of *p* < 0.001. The post-forest activity segment (segment 8) was also significantly different from all of the other segments, with *p* < 0.01; except for the breathing (segment 3) and pine-tree refuge (segment 7) segments.

Heart rate was highest during the breathing activity and lowest during the post forest activity segment. The breathing activity induced a significantly higher heart rate compared to all of the other segments (*p* < 0.001) and, following this activity, there was a downward trend of the mean HR. The mean of the median HR slopes showed no significant differences across the segments.

### 3.3. Ambient Temperature Has an Effect on Skin Temperature and Heart Rate Response

For the cool day’s participants, the average standard deviation of the EDA slopes and median of the skin temperature slopes were significantly higher following the Nature Break (*p* ≤ 0.001, *p* < 0.05). For the warm days’ participants, the mean average HR was significantly lower and the average median of the skin temperature slopes was significantly higher following the Nature Break (*p* = 0.001, *p* < 0.05).

Across the forest activity segments, participants from both the cool and warm days exhibited significant differences for all of the physiological features. The mean average HR was the highest during the breathing activity (segment 3) and demonstrated a generally decreasing trend following this segment for both the cool and warm day participants. This segment was significantly different from all of the other segments for the cool day’s participants and all of the other segments, except for the pre-forest activity segment, for the warm days’ participants.

The barefoot walking section (segment 5) induced the largest decrease in heart rate for the cool day’s participants and was significantly different from the sitting on stumps segment (segment 2) and the post-forest activity segment (segment 8) (*p* < 0.05); the latter two segments demonstrated the largest increase in heart rate. In contrast, the barefoot walking segment induced the largest increase in heart rate for the warm days’ participants and was significantly different from all of the other segments, apart from the post-forest activity segment (*p* < 0.05).

The cool day’s participants experienced a decrease in skin temperature during the pre-forest and post-forest activity segments (segments 1 and 8), whereas the warm days’ participants experienced the largest increase in skin temperature during these segments. Participants from all of the days experienced the largest or second largest decrease in skin temperature during the barefoot walking segment (segment 5). This segment was significantly different from all of the other segments for the warm days’ participants (*p* < 0.001), and all of the other segments, except the pre-forest, old tree, and post-forest activity segments (segments 1, 4, and 8), for the cool day’s participants (*p* < 0.05). These differences are consistent with the expected effects due to the diverging vasoconstriction and vasodilation response on hot and cold days, respectively.

### 3.4. Circadian Rhythms Affect Skin Temperature Response to Forest Bathing

The participants from all of the days demonstrated a significant difference between morning and afternoon groups for the change in average median of the skin temperature slopes pre-forest to post-forest (segment 1 to 8) (*p* ≤ 0.001). The afternoon had a smaller change in skin temperature slopes post-forest from pre-forest than the morning group. Cool day participants also saw a smaller difference in average heart rate post-forest from pre-forest in the afternoon group (*p <* 0.05). No other features demonstrated a significant difference between morning and afternoon groups in terms of pre-forest to post-forest differences.

Results of the ANCOVA showed no significant effect of time-of-day for the cool day’s participants, meaning there was no significant difference between morning and afternoon group post-forest when adjusted for the pre-forest measurements. For the warm days’ participants, the average median of the skin temperature slopes showed a significant effect of time-of-day (*p* = 0.001). The afternoon group experienced less of an increase in skin temperature post-forest than the morning group, with a significance of *p* = 0.001. The other features for the warm days’ participants showed no significant difference between morning and afternoon groups at the post-forest activity segment when adjusted for pre-forest measurements, pre-forest POMS scores, and time-of-day.

### 3.5. Participants Have Significant Physiological Differences Pre- and Post-Forest Bathing

**Figure 5:**
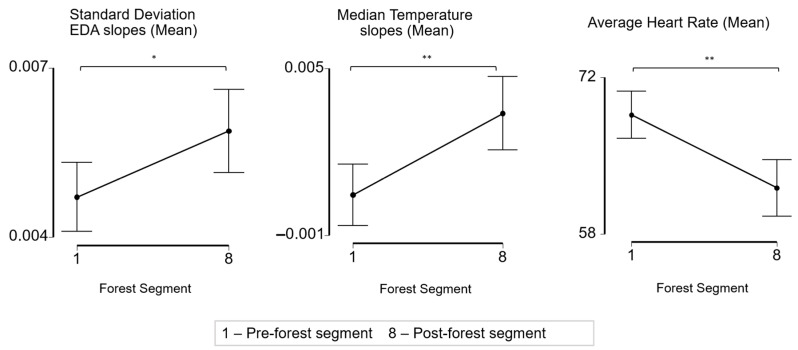
Effect of forest bathing on the mean standard deviation of the EDA slopes, mean of the median temperature slopes, and mean average heart rate from pre-forest activity segment to post-forest activity segment. * *p* < 0.05, ** *p* < 0.01, paired *t*-test and Wilcoxon signed rank test.

To facilitate the comparison of the results from this study to those of previous research, we also conducted a physiological analysis pre- and post-forest bathing. Across the participants, the average of the standard deviation of the EDA slopes and the median skin temperature slopes were significantly higher after the forest (*p* < 0.05, *p* < 0.01) and the average heart rate was significantly lower after the forest (*p* < 0.01) (Figure 5).

Notably, the post-forest physiological data included a segment where participants engaged in a discussion about their forest experience, in which several participants were visibly emotional. This may have confounded the post-forest physiological signals, resulting in the “post” segment not being representative of “post” intervention, but of a final active ingredient of the FB experience.

## 4. Discussion

The aim of this study was to explore the effects of an interactive, guided nature activity on physiological and psychological responses. Psychologically, the participants showed a significant decrease in their negative affect and a significant increase in their positive affect following a Nature Break. The physiological responses changed significantly according to the different activities within the forest, with participants also experiencing significant physiological differences before and after the Nature Break. Our results suggest that interactive nature activities have positive psychological benefits and demonstrate the feasibility of using wearable sensors to monitor physiological responses in a naturalistic forest bathing activity.

A growing body of evidence shows that FB and nature walks provide quantifiable physiological and mental benefits to human beings, such as reducing cortisol levels, HR, and blood pressure [3,5,12,14,48,49]. This study provides further evidence that an interactive nature activity increases positive mood states, decreases negative mood states, and has significant effects on physiological state. We found significant responses across all of the four physiological ANS features during the various activities of the Nature Break, indicating the potential for using a wearable sensor to monitor the effects of deliberate engagement with nature.

Previous studies using the POMS questionnaire found that negative mood states improved and positive mood states increased following a forest bathing and nature walk activity [4,8,11,49]. Other studies that have looked at purposeful engagement with nature have also reported mood enhancement and overall satisfaction with the nature session [29,30,31]. Our findings support this trend as well, providing further evidence that an interactive, guided nature activity can provide positive psychological experiences to humans. Cox, Shanahan, et al. (2017) deconstructed the nature experiences of 1023 residents of an urban population in the UK and found that a person’s connection to nature was positively correlated with them spending time in nature [50,51]. By fostering a positive forest experience, we anticipate that the Nature Break will similarly lead to participants spending more time in a forest environment in the future.

Few studies have assessed the effect of forest bathing in-situ using wearable sensors [25,49]. We showed that different activities conducted within the forest environment have different physiological effects, which contribute to creating a dynamic, embodied experience of immersion in nature. The participants across all of the days showed, in general, a decreasing average heart rate across the forest activity segments. These results are consistent with previous studies that have measured the effects of exposure to nature on heart rate [19,25,34,49,52,53]. EDA and peripheral skin temperature have received little attention in the context of FB and nature walks and studies that have investigated EDA did not report any significant findings [25,27]. Reeves et al. (2019) and Chen et al. (2018) have used EDA to assess ANS activity, specifically as a measure of physiological stress and arousal. Reeves et al. (2019) found no significant effects of site (wetland, control, urban) on tonic or phasic EDA. Chen et al. (2018) did not report specifically on EDA alone, but combined it with ECG measures to predict arousal and used HR and facial EMG to predict the valence. Chen et al. (2018) also measured skin temperature in their study, however they did not mention how it was used to contribute to their assessment of ANS activity. In contrast, we saw significantly greater standard deviation of the EDA slopes—indicating larger numbers of electrodermal reactions—and median of the skin temperature slopes post-forest compared to pre-forest across all participants.

Across the forest activity segments, the participants from all of the days had the largest sympathetic activity during the post-forest discussion (segment 8), old tree (segment 4), and barefoot walking (segment 5) segments, as manifested by their EDA, likely due to the various activities of touching, smelling, walking on dirt, and emotional discussion during these segments. As well, a lower standard deviation of the EDA slopes was observed during the pine tree refuge segment (segment 7), likely reflecting relaxation [38]. Peripheral skin temperature saw the greatest rate of decrease during the barefoot walking segment (segment 5) across all participants, likely reflecting the mental stimulation associated with focusing on the barefoot walking task [38]. Higher rates of increase in the peripheral skin temperature typically reflect mental relaxation and these were seen during segments such as the deep breathing, pine tree refuge, and post-forest activity segments (segments 3, 7, and 8) [38,43].

We found that the ambient temperature mainly had an effect on the skin temperature and heart rate response. The largest rate of decrease in the heart rate occurred during the barefoot walking segment (segment 5) for the cool day’s participants, however the warm days’ participants experienced the largest rate of increase during this segment. Similarly, the cool day’s participants showed a decrease in skin temperature during the pre-forest and post-forest section (segment 1 and 8), but the warm days’ participants had the largest increase in skin temperature during these segments. Differences in the rate of skin temperature change during the pre-forest and post-forest activity segments may be attributed to the difference in the ambient temperatures of those days and the presence of direct sunlight or overcast weather. The ambient temperature and other meteorological elements may also affect the ground temperature, which could have an impact on HR response during the barefoot walking segment [54]. In their study, Korhonen (2006) examined HR response in men who were exposed to local cold exposure and whole-body cold exposure. They found that local cold exposure to the feet increased the HR, while consistent whole-body exposure to cold lowered the HR [55]. Similar effects may characterize the difference in the HR responses in the cool day’s and warm days’ participants due to the variances in the air and surface ground temperatures.

Circadian rhythms also affected skin temperature. The skin temperature in the afternoon groups had a smaller change from pre-forest to post-forest. For the warm days’ participants, the afternoon groups’ skin temperatures increased less than the morning groups’ at the post-forest activity segment. The limited amount of meteorological data from this area limits our ability to explain this result as an effect of the changing ambient temperature throughout the day.

The results of this study need to be considered in light of several limitations. First, Venables and Mitchell (1996) suggest the importance of considering the interaction between the time of the day and the season with sex, as females may be more responsive to these conditions than men [56]. In addition to possible differences in physiological response between the genders, different physical environments may also induce varying psychological responses. Park et al. (2011) studied the relationship between various physical environments in forests and psychological responses; they found that they were significantly related [57]. This study did not account for the differences between the genders, nor other physical factors, apart from the average air temperature. Second, the participants were not required to perform the various exercises in the Nature Break. For example, some participants did not take off their shoes and socks to do the barefoot walking segment, which may have confounded the current results. Third, the wearable sensor would occasionally disconnect from the mobile application due to a Bluetooth malfunction. These intermittent disconnections accounted for several participants’ data being missing or too short to contribute to the analysis. Furthermore, when these moments occurred, a researcher had to intervene to check the equipment; this may have disrupted the complete immersive experience of the Nature Break activity. Fourth, there may be an effect of physical activity on the physiological responses in addition to the response from the forest bathing experience itself. Olafsdottir et al. (2020) investigated the effects of leisure walking in a natural environment against passive exposure to nature and physical exercise alone. They found that walking in nature improved mood more than just watching nature or doing physical exercise alone. They also found that, during a more stressful time period, walking in nature lowered cortisol levels more than nature viewing [48]. Hansen et al. (2017) also mentioned that multiple studies limited physical activities for each activity to 20 min in order to control for the effect of physical exercise on cardiovascular measures [3]. Although in this study we did not disentangle the effects of exercise on the physiological response from the effects of forest bathing, results from previous studies indicate that the act of walking may have contributed to the positive effects reported in our study.

Our study has shown that it is possible to use wearable sensors in order to monitor forest bathing experiences and this has significant implications for all organizations that aim to quantify the effect of nature on human physiology. Our results support using technology for a more evidence-based approach to forest bathing. They set the stage for future work that uses the distinct physiological response profiles that are associated with specific forest activities as a scaffold to guide the design of a forest immersion experience according to the intended outcome. Future work can also build upon the feasibility of using wearable sensors for moment-by-moment physiological monitoring in the forest to detect significant moments and activities for specific participants in real-time, allowing guides to tailor and customize the forest experience to the individual forest bather. 

## 5. Conclusions

We found that an interactive, guided nature activity increases positive mood states and decreases negative mood states. The various exercises in the forest had significant and varying physiological effects. In general, during the forest activity segments that involved more external stimuli that required focus and attention, such as walking barefoot in the forest or discussing personal experiences in the forest, the participants experienced a larger sympathetic activation; whereas, during the segments of introspection and relaxation, the participants experienced a larger parasympathetic activation. We found that there were differences in the physiological responses due to circadian rhythm, however we are not able to consistently report a main finding. For future forest bathing initiatives, we highlight the possibility of creating a program that is shaped by a target physiological response.

## Figures and Tables

**Table 1:** Description of segments in the Nature Break activity.

Forest Activity Segment	Description	Length (min.)
1: Pre-forest	The participants fill out pre-forest POMS and don the wearable sensor.	~10–20
2: Sitting on stumps	The UdN guide invites the participants to understand the course of time in forests and invites comparisons with the speed of life in the city.	~10–15
3: Breathing	The participants are standing. The UdN guide invites them to do a US Army breathing exercise and to focus on smells. The guide explains what scientists have found about certain particles emitted by trees and their healing benefits. S/he will also discuss facts about the air that is breathed in the city. The participants will practice a breathing exercise that they can repeat on a daily basis.	~10–15
4: Old tree	The participants are invited to lie down directly on the ground. The UdN guide explains the scientific discoveries that are related to the body’s contact with the ground. The participants practice an exercise that they can repeat on a daily basis. The participants are also invited to grab a handful of dirt and to observe and smell the contents.	~20
5: Walking barefoot	The participants are invited to remove their shoes and socks for the duration of the walk to the next stop.	~5
6: Ferns	The participants are invited to observe and listen to their immediate environment. The UdN guide explains Dr. Ulrich’s “View through the window” study [35] and the uses of nature in hospitals since this discovery. The participants practice an exercise that they can repeat on a daily basis.	~20–30
7: Pine tree refuge	The guide explains the discoveries that demonstrate the positive impact of nature on mental health and invites participants to find their own refuge in this clearing. They stay here for a couple of minutes and then meet the guide. The participants are invited to taste some resin from the pine trees.	~10–15
8: Post-forest	the guides lead a group discussion about the participants’ forest experience. The participants fill out post-forest POMS.	~15–20

## Data Availability

The physiological and POMS data that were used to support the findings of this study are available from the corresponding author upon request.
